# An Unusual Case of Kyrle Disease

**DOI:** 10.7759/cureus.67767

**Published:** 2024-08-25

**Authors:** Sanjeev Gupta, Reshma Gupte, Rohan Manoj, Asharbh Raman, Yash Buccha

**Affiliations:** 1 Dermatology, Dr. D. Y. Patil Medical College, Hospital and Research Centre, Dr. D. Y. Patil Vidyapeeth, Pune, IND

**Keywords:** kyrle disease, dapsone treatment, acitretin, asymptomatic hyperuricemia, acquired perforating dermatoses

## Abstract

Kyrle disease is one of the acquired perforating disorders (APDs), commonly associated with type 2 diabetes, chronic kidney disease, and other pruritic conditions. Here, we report a case of Kyrle disease with characteristic transepidermal elimination of dermal contents on histopathology. However, the only abnormal laboratory finding in our patient was hyperuricemia, and none of the commonly associated underlying conditions were found. The precise etiopathogenesis of APD is poorly understood. Abnormal glycation of collagen in diabetes and defective follicular keratinization following chronic friction have been commonly implicated in the pathogenesis. Treatment of the underlying cause is the mainstay; however, our patient responded well to acitretin, dapsone, and topical corticosteroids. Further studies need to be done to evaluate other possible causes, such as hyperuricemia in our patient, especially when none of the classical associations are present after a detailed workup.

## Introduction

Acquired perforating disorders (APDs) are a heterogeneous group of four disorders, namely, Kyrle disease, perforating folliculitis, acquired elastosis perforans serpiginosa (AEPS), and acquired perforating collagenosis (APC). These disorders are often associated with pruritis, erythematous-to-violaceous papules, and keratin plugs. These disorders have subtle differences in age of onset, gender predilection, associated comorbidities, and clinical features. However, their similarities far outweigh their differences, compelling most researchers to describe them under the umbrella term of APD [1]. Diabetes mellitus, hemodialysis, and renal failure are the most common associations, especially with Kyrle disease. However, it can also occur secondary to pruritic conditions such as insect bites, scabies, and hepatobiliary diseases, often labeled as a form of prurigo nodularis [1,2]. Although the precise etiopathogenesis is not known, superficial trauma to the epidermis seems to be the most likely trigger owing to its high association with pruritic dermatoses [2]. Interestingly, APD often involves the most accessible sites such as extremities where patients can scratch easily, leading to koebnerization and progression of lesions [2]. A history of trauma and intractable pruritus at the onset of lesions, coupled with the dissemination of the lesions over sites of new trauma, led experts to believe that microtrauma might play an essential role in the pathogenesis of the disease [2]. Microtrauma can lead to the formation of defective collagen degradation products in genetically predisposed individuals, which can subsequently facilitate their transepidermal elimination. Furthermore, the disease process often improves when the patient is prescribed antipruritic medications, breaking the itch-scratch cycle and reducing microtrauma [2]. Here, we present an unusual case of Kyrle disease with no underlying systemic associations.

## Case presentation

**Figure 1:**
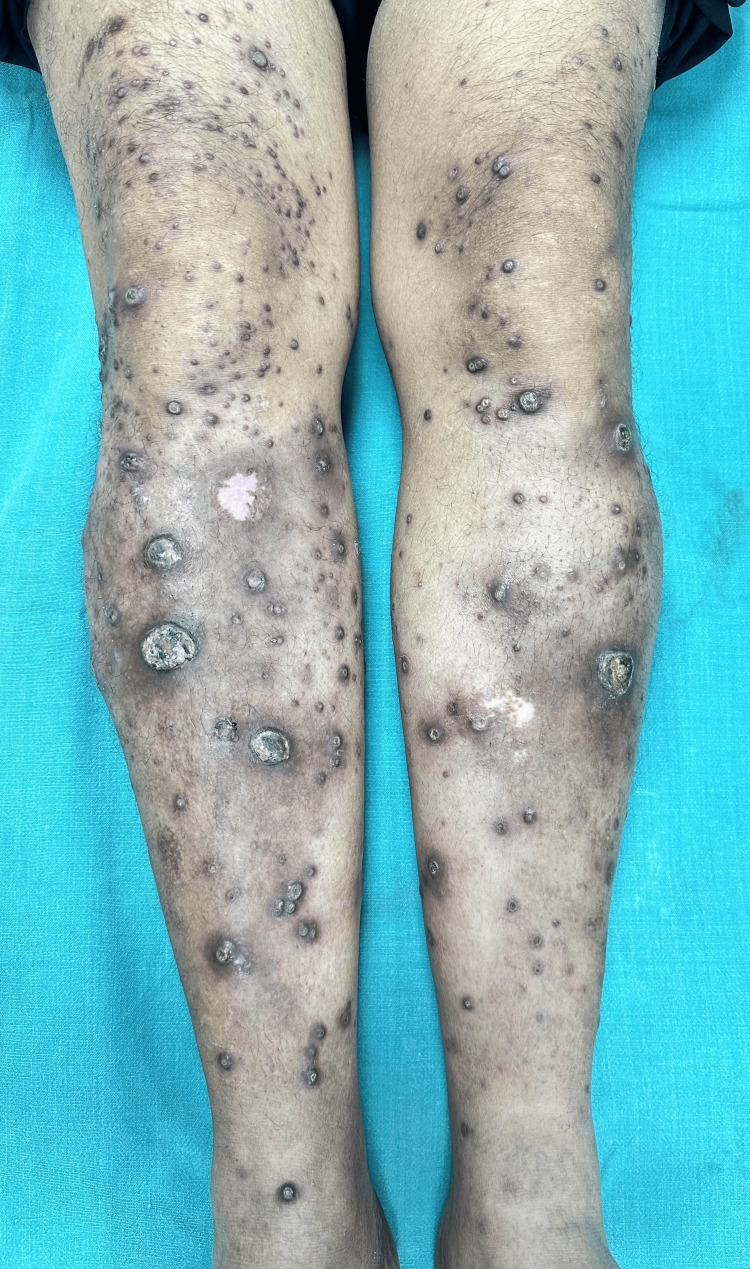
Multiple erythematous hyperkeratotic papules to plaques and few crateriform erosions with central hyperkeratotic crust, associated with post-inflammatory hyperpigmentation present over the bilateral lower limbs.

A 54-year-old man presented with intermittently pruritic, hyperpigmented-to-violaceous papules and pustules over the extensor surface of the legs (Figure 1). The lesions began as small erythematous papules, progressing to form large, crusted plaques over a five-month period. The patient confirmed a history of new lesions appearing at the sites of previous trauma and excoriations due to repeated scratching, progressing to form crateriform erosions which subsequently healed with scarring. Nails and oral mucosa were unaffected. His serum uric acid was raised (8.8 mg/dL). However, blood sugar, HbA1c, thyroid profile, and liver and renal function tests were normal. Antinuclear antibodies were not detected on immunofluorescence. Ultrasonographic examination of the neck and abdomen yielded no aberrations. On inquiry, he denied any previously diagnosed comorbidities or a history of recent medication before the onset of the lesions.

**Figure 2:**
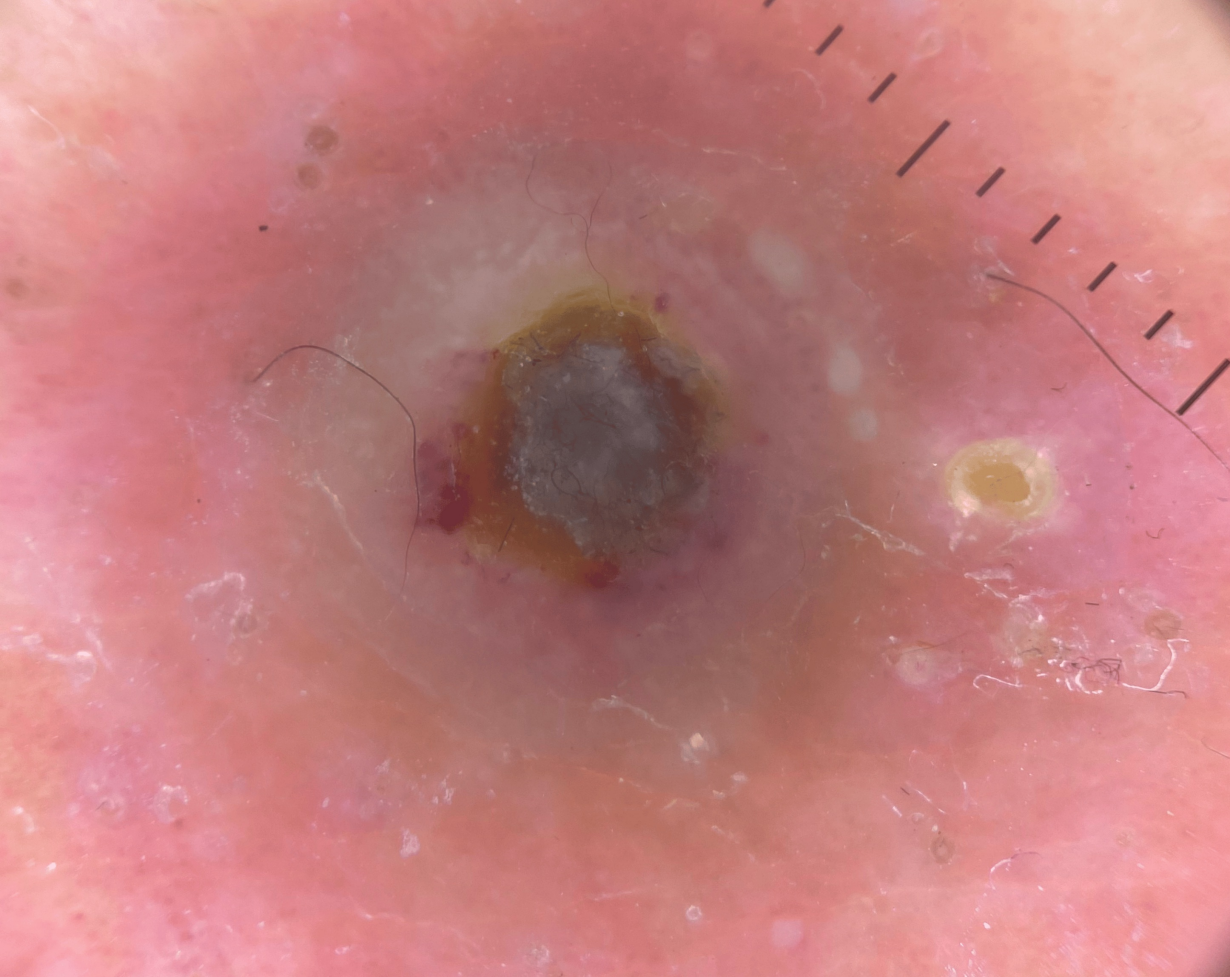
Dermatoscopy showing a central, yellowish-brown, structureless area surrounded by scaling and linear and dotted vessels, with an irregular white ring at the periphery.

**Figure 3:**
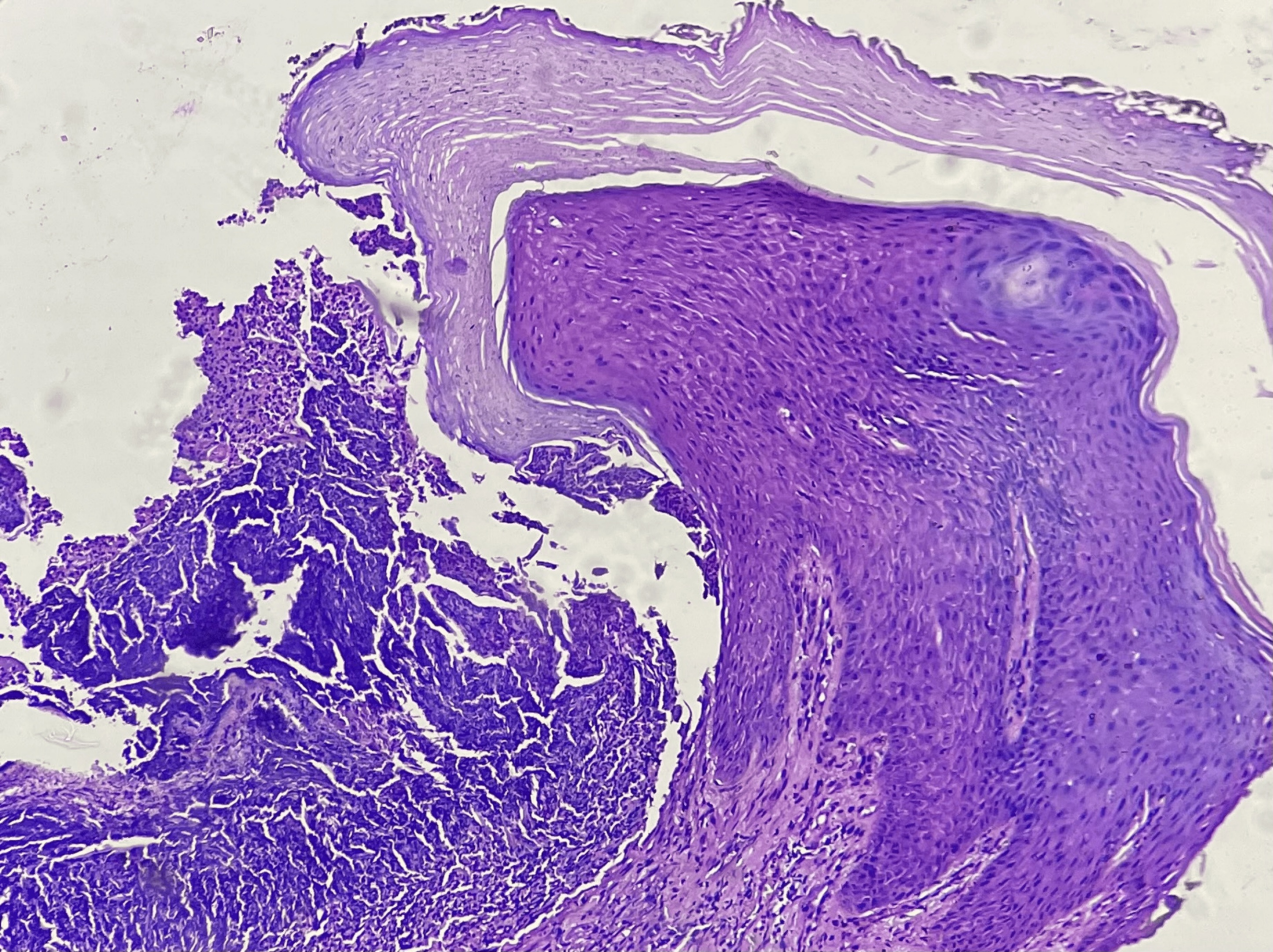
Histopathological examination showing cup-shaped invagination of the hyperkeratotic epidermis with degenerated dermal collagen, elastin, and inflammatory cells being eliminated transepidermally.

A dermatoscopic evaluation revealed a yellowish-brown structureless area surrounded by scaling and linear and dotted vessels, with an irregular white ring at the periphery (Figure 2). Histopathological examination showed a cup-shaped invagination of the hyperkeratotic epidermis, with degenerated dermal collagen, elastin, and inflammatory cells being eliminated transepidermally (Figure 3).

Both APD and prurigo nodularis were considered as possible diagnoses. Prurigo nodularis may present similarly with no evidence of any underlying pathology. However, the presence of erythematous papules and pustules with follicular plugs, along with crusted plaques, made this diagnosis highly unlikely. Based on the clinicohistopathological findings and dermatoscopic evaluation, a diagnosis of Kyrle disease was made [3,4]. The patient was started on oral acitretin 25 mg once daily, and dapsone 100 mg once at night, along with potent topical steroids and urea-based emollients, followed by considerable symptomatic improvement over the next two months.

## Discussion

Kyrle disease is one of the uncommon APDs, often associated with an underlying systemic comorbidity [5]. Primary APD is often associated with chronic debilitating diseases, such as diabetes and chronic kidney disease (CKD) [1,2]. CKD in itself is most commonly secondary to diabetic nephropathy, suggesting some overlap between both underlying associations [1]. Less commonly, APD can also be secondary to rarer conditions such as perforating granuloma annulare, calcinosis cutis, and keratoacanthoma [2]. However, in our patient, no underlying cause could be identified, except for raised serum uric acid levels. Raised uric acid levels have been associated with APD according to a few studies [2]. However, many studies do not mention it specifically as a cause of APD unlike the common associations such as diabetes and renal failure [3].

Transepidermal elimination of dermal components such as collagen and elastin is a hallmark feature of APD on histopathology [4]. Interestingly, patients present with polymorphic lesions at different stages of the disease progression. Hence, there may be immense variability between the histopathological findings between an early lesion and a late lesion of the same disease in a particular patient. Furthermore, the histopathological similarities between the four groups of disorders included under APDs make the dermatopathological extremely challenging. Good history-taking and clinical examination can help differentiate among APC, AEPS, perforating folliculitis, and Kyrle disease. APC and AEPS often present with a family history and can occur at a younger age [6]. Furthermore, the annular lesions of AEPS are quite distinctive compared to the other three APDs. AEPS is also associated with a myriad of other unique genetic disorders that often target collagen and elastin formation such as Marfan syndrome, Ehlers-Danlos syndrome, osteogenesis imperfecta, pseudoxanthoma elasticum, and Rothmund-Thomson syndrome, to name a few. Kyrle disease has both follicular and parafollicular plugs, unlike perforating folliculitis, and is more often associated with Koebner’s phenomenon [1,6]. Sometimes, APD can develop secondary to drug exposure. Penicillamine can cause AEPS, while tumor necrosis factor inhibitors, epidermal growth factor inhibitors, antivirals such as indinavir and sirolimus, and vascular endothelial growth factor inhibitors such as bevacizumab can also be a cause of APD [6]. Dermatoscopic examination can be invaluable in ruling out other differential diagnoses such as lichen planus and prurigo nodularis. APD lesions can often present with yellowish-brown structureless areas in the center over an erythematous background with radially arranged vessels in the periphery and a rim of peripheral hyperpigmentation [3]. The precise etiopathogenesis of APD is poorly understood. Maillard adducts to connective tissue products formed due to non-enzymatic abnormal glycation of collagen and elastin in patients with diabetes offer a possible explanation for the pathogenesis of the disease. Additionally, in patients with CKD, improper renal clearance of these Maillard adducts may contribute to the risk of developing APD even further [2]. Pruritus and microtrauma may facilitate the transdermal elimination of these altered connective tissue elements. Defective follicular keratinization following chronic friction has also been linked with the pathogenesis [2,5]. Recently, a few additional theories regarding the pathogenesis of APD have been hypothesized. There is epidermal proliferation and extension into the dermis following chronic pruritus. This hyperplastic epidermis eventually surrounds the abnormal dermal components similar to a foreign body reaction and facilitates its transdermal elimination. Another recent theory involves the initial formation of a hyperkeratotic plug at the base of the epidermis, which penetrates this intact epidermis and begins the cascade of processes involved in APD [6]. Plasma values of fibronectin are often raised in diabetes. Interestingly, fibronectin is often concentrated locally in lesional sites of APD. This led many researchers to believe that this aberrant fibronectin may be forming complexes with type IV collagen. These complexes may stimulate epidermal hyperplasia and perforation [6].

Corticosteroids and retinoids, in both topical and systemic formulations, are the mainstay of treatment. There is also evidence that doxycycline, dapsone, methotrexate, and hydroxychloroquine can hasten recovery [7-9]. APD lesions often get secondarily infected with *Staphylococcus aureus* leading to a pustular discharge. Hence, antibiotics such as doxycycline may be beneficial to treat these acute infective complications. A recent systematic review mentioned allopurinol as a first-line treatment option, owing to its property as a free radical scavenger. They mentioned the successful use of the drug in several case reports and series published previously, raising the possibility of hyperuricemia being an undetected underlying condition that responded well to allopurinol [7]. These diseases are often recalcitrant and do not respond unless the underlying comorbidity is adequately controlled [8-10]. A thorough workup failed to reveal any underlying conditions in our patient except for hyperuricemia. However, he showed good symptomatic improvement with a combination of acitretin and dapsone orally as well as potent topical corticosteroids and urea-based emollients. Rarely, there can be a few cases of APD such as our patient without any underlying comorbidities [11].

## Conclusions

A vast majority of the patients with Kyrle disease have an underlying comorbidity such as diabetes, chronic renal failure, or other pruritic conditions. In rare instances, clinicians fail to detect any of the known associated conditions, as seen in our patient. It is crucial to consider other differentials in these cases such as prurigo nodularis, lichen planus, and other pruritic conditions which are not often associated with any underlying pathology. Dermatoscopic evaluation and histopathological examination are invaluable in diagnosing APD, as seen in our patient. Hyperuricemia seen in our patient was the only abnormal laboratory finding. Whether this was an unrelated coincidence or played a role in disease pathogenesis needs to be evaluated with further studies, as a significant number of patients have been successfully managed with allopurinol, a known uric acid-lowering drug.
